# Coevolution Dynamics of Beneficial and Pathogenic Microbes in Plant–Microbe Interactions

**DOI:** 10.3390/biology14111505

**Published:** 2025-10-28

**Authors:** Afeez Adesina Adedayo, Mary Tomi Olorunkosebi

**Affiliations:** Department of Biological Sciences, Western Illinois University, Macomb, IL 61455, USA

**Keywords:** microbial arm race, endophytes, holobionts, rhizosphere microbiomes, sustainable agriculture

## Abstract

**Simple Summary:**

This review investigates the ways in which plants and the microbial populations that surround them coevolve to affect resilience, growth, and health. It draws attention to the selection pressures that both pathogenic and beneficial microbes exert on plants and one another, causing genetic and physiological changes that influence interactions in the endosphere and rhizosphere. The study highlights the effects of endosymbiosis, mutualism, and antagonism on plant fitness and agricultural yield as important microbial coevolution processes. The translation of laboratory results to field systems, the long-term effects of climate change, and our inadequate grasp of intraspecific microbial diversity are among the major knowledge gaps. According to the review’s findings, innovations based on microbiomes, including resilient cropping systems, precision biofertilizers, and coevolutionary breeding, can be guided by the idea that plants are holobionts.

**Abstract:**

The intricate connections between plants and the microbial populations that surround them are crucial for plant development and resilience, but little is known about the evolutionary processes influencing these partnerships. Less is known about how pathogenic and beneficial microbes coevolve with their plant hosts over ecological and evolutionary timeframes, despite the fact that several studies identify rhizosphere and endophytic microbes that support nutrient acquisition, disease resistance, and stress tolerance. Using molecular, ecological, and evolutionary investigations from soil, rhizosphere, and endosphere habitats, this review summarizes current findings on microbial coevolution in plant–microbe systems. We look at the endosymbiotic processes that underlie the development of organelles, the mechanisms of mutualism and antagonism, and the eco-evolutionary feedbacks that affect plant health and agricultural output. The inadequate comprehension of intraspecific microbial diversity, the application of laboratory coevolution experiments to field settings, and the long-term effects of climate change on the evolutionary dynamics of plants and microbiomes are some of the major knowledge gaps. When pathogenic and beneficial microbes apply selective pressures to one another and their common host, coevolution takes place. This results in mutual genetic and physiological adaptations, such as modifications to host immunity, microbial virulence, or competitive tactics, which influence the way the two types interact over time. We conclude that understanding plants as holobiont-integrated units of hosts and their microbiomes offers fresh chances to develop microbiome-based approaches to sustainable agriculture, such as coevolutionary breeding programs, precision biofertilizers, and resilient cropping systems.

## 1. Introduction

The soil around the root region of a plant with a depth of 4–15 cm is called rhizosphere soil [1]. The soil region is an area of microbial diversity, which includes bacteria, fungi, archaea, nematodes, and other microbes that carry out beneficial activities to the plants during plant–soil interaction [2]. Some of the major functions carried out by these microbes include carbohydrate metabolism, plant growth promotion, nutrient cycling, disease resistance abilities, and resilience to abiotic stress (drought, extreme pH, cold, etc.) [3]. Aside from rhizosphere soil colonization, microbial communities can be found in the tissues of plants, and they are called endophytes [4]. They are found in plant roots, stems, and branches. During the interaction of plant roots and soil microbes, the plant roots produce some organic substances called root exudates. These organic materials produced attract the soil microbes into the rhizosphere region [5]. They feed on these carbon substrates and are accommodated in the rhizosphere region of the plant to enable microbe–plant signaling. To return the favor, the microbes carry out various functions listed above in the region of the plants, which results in a mutual relationship called symbiosis [3].

Through the use of pattern recognition receptors (PRRs), the innate immune systems of plants distinguish between beneficial and pathogenic microorganisms. Immune responses that limit infection are triggered by pathogen-associated molecular patterns, or PAMPs. On the other hand, beneficial microorganisms avoid significant immune activation by producing altered or repressed signals. Additionally, microbe- or symbiosis-associated molecular patterns (MAMPs/SAMPs) that encourage mutualistic relationships are recognized by plants. Plants adjust their defensive and symbiotic responses for maximum growth and safety by striking a balance between recognition and tolerance [6].

Accordingly, these organisms are manipulated through agricultural practices to contribute to plant health. Plant health status is supported and enhanced by symbiotic relationships with the soil microbes as well as endophytic microbes.

These manipulations are supported by ecological-theory-based co-evolution [7]. A typical example is observed in ecological niches and the competition that occurs between phytopathogens and beneficial microbes, where beneficial microbes inhibit or absolutely eradicate the existence of pathogenic microbes, thereby keeping the plant healthy. The existence of abundant microbial diversity in the soil or the tissue of the plants contributes to rhizosphere ecosystem functioning. It reveals the potential of the community that is determined by its productive members, a process called selection effects, and the other effect is a condition referred to as intrinsic advantage, observed by the productive members, otherwise called complementary effects [8]. The beneficial inter-relationships occurring between microbial species and functioning ecosystems [9,10] have been reported in various studies monitoring their significance within specific groups of species (intraspecies), despite the importance of interaction between plants and microorganisms and host-related microbe functions. Furthermore, referring to microbial communities as the raw materials and product of natural selection, intraspecific microbes are recognized for their prevalent functions in the process of co-evolution involving plant–microbial interaction, as reported by Mesny et al. [11].

The coevolution of terrestrial ecosystems results from the organization of microbes, including bacteria, fungi, archaea, etc., on or in plants, which is regarded as an immense phase [12]. However, the exploration of these microbial communities associated with and empowering one another has not been investigated. Fossil evidence has revealed the inter-relationship among microbial communities and bryophytes [13]. These microorganisms were reported to be involved in processes including the weathering of rock, formation of soil, and sediment stabilization, which eventually result in ecosystem richness [14]. While plants are growing in the soil, microbial functions manifest, among which are their abilities to improve plant tolerance against living (biotic) and nonliving (abiotic) stresses [15]. These microbes undergo symbiotic relationships as observed in arbuscular mycorrhiza (AM) and plant-growth-promoting rhizobacteria (PGPR) interaction that promotes biodegradation of organic molecules [16], production of phytohormones, nutrient acquisition, microbe–plant signaling, biological control of phytopathogens, induced systemic resistance (ISR), and other beneficial functions to the plant [2]. Plants, on the other hand, produce exudates—carbon substrates that serve as food for the microbes and also house the phytomicrobiomes [17]. Furthermore, the plant and microbial communities’ interaction leads to the formation of a holobiont.

Although plants are holobiont-integrated organisms made up of a host and its surrounding microbiota [18], little is known about the evolutionary processes forming these alliances. The main issue posed by this review is how pathogenic and beneficial microbes coevolve with their plant hosts, and how this knowledge is used to increase the resilience and sustainability of agricultural systems. We postulate that the stability of mutualistic connections and the development of plant defenses against diseases are driven by reciprocal evolutionary change between plants and their microbial partners, which in turn affect crop yield and ecosystem function. Rapid developments in high-throughput sequencing, experimental evolution, and multi-omics analysis during the last five years have shown remarkable patterns of microbial diversity and adaptation in the endosphere and rhizosphere of plants. Abundant microbiome diversity [2] can reduce pathogen prevalence and improve nutrient cycling in outdoor settings, according to studies employing worldwide surveys. Phage–bacteria dynamics studies emphasize how antagonistic coevolution shapes nutrient fluxes and rhizosphere communities [19]. According to multi-omics research, certain plant genotypes attract different microbial consortia, indicating that host and microbiome co-selection is continuous [20]. However, there are still important knowledge gaps: the ecological significance of intraspecific microbial variation is still poorly understood; laboratory coevolution studies frequently fall short of capturing the complexity of field environments; and the long-term evolutionary effects of climate-driven stress on plant–microbiome interactions are not well understood.

This review focuses on the coevolution of microbial communities and their functions in relation to plant species, emphasizing the significance of microbial coevolution and evolutionary natural selection in plant–microbe interactions within the rhizosphere. It explores endosymbiotic interactions that have led to the coevolution of specific plant organelles and highlights fundamental microbial roles in plant growth promotion, survival, and competition between beneficial and phytopathogenic microbes. The study proposes that incorporating the holobiont concept into agricultural practices can enhance microbiome diversity in soil and plant tissues for sustainable agriculture. Furthermore, it offers a fresh synthesis by explicitly framing plant–microbe interactions within a coevolutionary and eco-evolutionary context, unlike previous studies that focused mainly on plant-growth-promoting rhizobacteria (PGPR), fungi (PGPF), or microbial roles in nutrient acquisition. By integrating insights from community ecology, applied agronomy, and molecular evolution, this review argues that understanding reciprocal evolutionary dynamics is essential for developing microbiome-based innovations such as precision biofertilizers and breeding strategies that promote resilient holobionts in the face of climate change [2].

## 2. The Concept of Microbial Coevolution

The process by which two or more interacting species mutually impact one another’s evolutionary trajectory over successive generations is known as coevolution [21]. This dynamic in plant–microbe systems includes both antagonism, such as arms races between crops and bacterial or fungal diseases [22], and mutualism, such as rhizobacteria that promote plant development [2] and arbuscular mycorrhizal fungi [6]. The host plant, its rhizosphere and endophytic microbiota, related viruses, protists, and even mobile genetic elements make up the plant holobiont, a conceptual unit in which these interactions take place [4]. The microbiome is an essential part of plant fitness and evolution, not just a group of temporary colonists. Coevolution can occur pairwise, as in host–pathogen gene-for-gene dynamics, or diffusely, as in networks of interacting taxa [23]. Understanding the distribution of selection forces in intricate soil and plant ecosystems requires the ability to distinguish between various modalities.

### 2.1. Molecular Mechanism of Coevolution

Rapid microbial coevolution and well-calibrated molecular rate signaling facilitate reciprocal adaptability. The microbial consortia in the rhizosphere are shaped by the variety of sugars, amino acids, organic acids, and secondary metabolites that are exuded by plant roots [24]. These substances serve as carbon sources and chemoattractants. In order to take advantage of these exudates, microbes develop receptors and catabolic pathways, generating feedback loops that strengthen certain alliances. Plants use pattern-recognition receptors (PRRs) at the immunological interface to identify molecular patterns (MAMPs/PAMPs) linked to beneficial and pathogenic microbes [25]. In a typical Red Queen hypothesis, infections develop new effectors to get past plant resistance genes, whereas beneficial bacteria can avoid or inhibit these reactions through secretion systems, effector proteins, or short RNAs [26]. Through plasmids, integrative elements, or bacteriophages, horizontal gene transfer speeds up microbial adaptability and makes it possible for characteristics like antibiotic resistance or production to spread quickly [27]. The endosymbiotic origins of mitochondria and chloroplasts continue to be the quintessential case of plant–microbe coevolution over an evolutionary period, illustrating how long-standing symbioses may irreversibly alter host metabolism and genomic structure [28].

### 2.2. Coevolutionary Trend of R-Gene/Effectors or Mycorrhizal Signaling Coadaptation

Plants acquire resistance (R) genes that produce immune receptors that can recognize specific pathogen effectors or molecules released by microorganisms to inhibit plant defenses, a process known as R-gene/effector coevolution [29]. In order to avoid detection, pathogens constantly alter or swap out these effectors, which fuels an evolutionary arms race. The genetic variety of microbial effector repertoires and plant R-genes is preserved by this Red Queen hypothesis [30]. One example of this ongoing molecular tug-of-war is the coevolution of *Pseudomonas syringae* effectors with *Arabidopsis thaliana* R-proteins [31]. On the other hand, rather than being driven by conflict, mycorrhizal signaling coadaptation represents a mutualistic coevolutionary tendency [32]. To create nutrient-exchange symbioses, plants and AM fungus have developed highly conserved symbiotic signaling pathways, such as Myc factors (fungal lipochitooligosaccharides) and plant receptors like LysM-domain receptor kinases. This has resulted in improved molecular compatibility over evolutionary time, allowing fungi to acquire carbon and plants to absorb phosphorus efficiently [33].

### 2.3. Ecological Evidence from Natural and Experimental Systems

The extent to which these molecular processes scale to communities and ecosystems is becoming more and more apparent via field and lab research. Abundant rhizosphere microbiome diversity reduces pathogen incidence and enhances nutrient cycling in a variety of climates and soil types, according to global studies employing shotgun metagenomics [5]. Negative frequency-dependent selection, which preserves effector diversity and permits coexistence with resistant plant genotypes, is demonstrated by long-term monitoring of *Pseudomonas syringae* populations [34]. Antagonistic coevolution between bacteria and bacteriophages is highlighted by experimental evolution in soil microcosms [35]. This coevolution modulates nitrogen and carbon fluxes across the rhizosphere and accelerates the turnover of microbial lineages [36]. Bacterial defensive features, such as biofilm formation and secondary metabolite release, are selected for by protists like amoebae, which also happen to improve plant growth promotion and disease suppression. However, it is still difficult to apply these findings to field agroecosystems because seasonal variations, environmental variability, and management techniques (such as fertilization and tillage) provide layers of selection that are not present in controlled studies.

## 3. Microbial Communities’ Co-Evolution in Plant–Microbial Interaction

Natural selection is a key factor in causing speciation and diversity through microbial mutation and recombination (Table 1). Rapid microbial coevolution in laboratory environments provides information on microbial adaptability to environments like plant rhizospheres [37]. Significant bacterial diversity and the coevolutionary dynamics between bacteria and their viruses, bacteriophages (phages), have been emphasized by studies examining soil microcosms with and without plants [38]. The cooperative adaptation of bacteria and phages illustrates how peripheral species influence microbial coevolution within specific environments. However, studies on the interactions between plants and their rhizospheres frequently ignore intraspecific variation in favor of microbial richness, diversity indices, and abundance as determined by sequencing techniques like Sanger (amplicon) sequencing [39].

### 3.1. Endosymbiotic Coevolution

Eukaryotes, i.e., organisms with true nuclei, were believed to coevolve from prokaryotes, i.e., organisms with no nuclei, via certain processes. The first process includes the evolution of the prokaryotic microbes that are aerobic. Within fungi (eukaryotes), several bacteria (prokaryotes) and viruses can form symbiotic partnerships that benefit their fungal hosts in a number of physiological and ecological ways. For instance, the endosymbiotic bacteria *Burkholderia rhizoxinica*, which lives in the fungus *Rhizopus microsporus*, increases the fungus’s competitive fitness by producing rhizoxin [49], a strong toxin that causes the fungus’s pathogenicity in rice seedlings. Similarly to this, mycoviruses like *Cryphonectria hypovirus* 1 (CHV1), which infects the fungus that causes chestnut blight, *Cryphonectria parasitica*, can reduce fungal virulence and increase host survival [50], which indirectly helps the fungus maintain ecological balance and long-term persistence. In the end, these intracellular connections can help fungi adapt to a variety of environmental niches and build more robust ecological partnerships by influencing fungal metabolism, stress tolerance, reproduction, and host–pathogen interactions. The coevolution includes the development of protomitochondrions and their ability to live in an oxygenated environment [28]. An endosymbiotic proteobacterium is incorporated through the eukaryotic evolution descending through mitochondria [51]. The first amitotic amoeboid, which is an aerobic organism, comes into existence from the obligatory endosymbiotic association through coevolution [28]. The facts about endosymbiosis have been under investigation for decades in molecular evolution and cell biology, which have revealed how mitochondria originated, resulting in the divergence of eukaryotic organisms [52]. This shows that living eukaryotes or their origin lineage should possess mitochondria, but they have been removed during host–parasite association. Other essential steps in the coevolution of plants were the endosymbiosis of cyanobacteria, synthesizing sunlight, which led to the formation of chloroplasts due to the abundance of gene loss from the cyanobacterial symbionts that occurred during the chloroplast coevolution, which is the major retained functional aspect that has been kept [53].

Selective benefits are always available for stability in the coevolution of the endosymbiontic association. The duo involved in the relationship is selected for groups that reproduce individually. Cells originating from different ancestries must have coordinated their potential for co-evolutionary advantages and produce activities supported by their contradictory features to accomplish stable endosymbiosis. The functions of chloroplasts and mitochondria are essential for plant growth promotion. Chloroplasts are used by plant cells to produce chlorophyll in plants’ green leaves to trap sunlight during photosynthesis. During the process, CO_2_ is also assimilated by plants to generate glucose and oxygen molecules released as by-products. Furthermore, there is a synthesis of amino acids and fatty acids, a reduction of nitrite to ammonium, and sulfate uptake for organic material incorporation. Mitochondria are utilized by plants and animal cells for cellular respiratory purposes. They use electron acceptors as substitutes to produce ATP, thereby transporting it to other parts of the organism’s body for metabolic activities. It is obvious that these organelles essentially coevolved from microbial communities and are regarded as the basic means of plant evolution [54]. The existence of plastids and mitochondria displays an organism’s genetic makeup since the genes embedded in these organelles are responsible for genomes and expression obtained from the bacterial phyla, including cyanobacteria species [55]. Analyzing the DNA sequence of plant tissues reveals various similarities between bacterial species as reported by Öztürk and Kayaaslan [56]. This further reveals why it is challenging to conduct amplification of bacterial genes from plant tissues. Nevertheless, studies have shown that the knowledge gap has been bridged between plants and phytomicrobiomes, which constitute the holobionts that promote the association in the coevolution of microbial communities with the plants [28].

#### Symbiotic Nodule Microbes and Their Coevolution with Plants

Leguminous plants and symbiotic nodule bacteria, or rhizobia, have coevolved to create highly specialized mutualistic associations that support biological nitrogen fixation [57]. The complex molecular signaling between the bacteria and plant roots is the first step in this coevolution [58]. Flavonoids released by the plant cause bacterial Nod genes to be expressed, which results in the synthesis of Nod factors that cause the curling of root hair and the development of nodules [29]. Bacteria like *Rhizobium leguminosarum*, which is linked to peas (*Pisum sativum*) and clover [59]; *Sinorhizobium meliloti* and *Priesta aryabhattai*, which are linked to alfalfa [60]; *Bradyrhizobium japonicum*, which is linked to soybeans [61]; and *Mesorhizobium loti*, which is linked to Lotus species [62], differentiate into bacteroids inside these nodules. These bacteroids transform atmospheric nitrogen into ammonia, which is used by plants for growth. This collaboration has resulted in host-specific adaptations over evolutionary time, with bacteria improving their symbiotic effectiveness and host range and plants developing selective recognition mechanisms for suitable rhizobia [63]. This kind of coevolution is essential to sustainable agriculture because it improves soil fertility and nitrogen availability while lowering reliance on artificial fertilizers.

### 3.2. Antagonistic Coevolution in Plant–Microbe Interactions

The biological control interaction between the microbes inhabiting the rhizosphere or between the microbes and plants is the same as the symbiotic association. The coevolution of the competitive association between the microbes could result in indirect consequences on plants through the composition and ecosystem functions of the rhizosphere microbial communities [64]. Beneficial and pathogenic microbes co-evolve through continuous reciprocal adaptations as they interact with their shared plant hosts. Pathogens evolve mechanisms to overcome plant defenses and establish infection [65], while beneficial microbes develop strategies to evade or suppress host immunity and promote mutualistic associations [66]. In response, plants refine their immune recognition systems to distinguish friend from foe, driving further microbial evolution [67]. This dynamic interplay of attack, defense, and counter-defense shapes microbial diversity, influences community composition in the rhizosphere, and maintains a balance between disease suppression and plant growth promotion over evolutionary time. However, some chemical substances have been used to control diseases caused by phytopathogen invasion, resulting in competition that is phytotoxic, i.e., toxic to plants, thereby depriving plants of their survival. The competition among microbial communities for food substances and spaces can indirectly mediate and mostly reduce the microbial population. Moreover, microbial contact-dependent or -independent interaction does occur via direct mediation. This mediation brings about how the coevolution of microbial communities’ competition rapidly occurs in the rhizosphere, which is not well understood. Some indirect studies propose that species of bacteria, including *Streptomyces* spp. and fungi species such as *Fusarium oxysporum,* can adapt and reveal antagonism during the co-occurrence of their co-evolutionary history. The research conducted on microbial transplantation from their immediate ecosystem to another revealed that *Curtobacterium* does well with adaptation to their immediate environment via genetic mutations of microbial functional diversity, including stress response, production of exopolysaccharides, and acquisition of nutrients [68]. This displays how bacteria actively coevolve as a result of changes in the composition of the microbial community within a limited time interval. Meanwhile, competition among microorganisms could have a positive and indirect effect on the health status of the growing plants through phytopathogenic competition with plants that can accommodate spoilage organisms’ invasion as a result of the inability to fight off the phytopathogens.

The interaction of plants and PGPR prevents parasitic microbes from affecting plants. A typical example is observed in the *Solanum lycopersicum* study as presented by [5], which displayed how *Oidium neolycopersicum*, a fungal agent causing powdery mildew of tomato, is controlled by beneficial rhizosphere microbes. Omomowo, et al. [69] claimed that *Trichoderma viride* and *Penicillium chrysogenum* obtained from the rhizosphere of *Moringa oleifera* biologically control soft rot disease of *Citrus sinensis* caused by *Aspergillus niger, A. citri, Fusarium oxysporum,* and *Penicillium digitatum*. The interaction between the beneficial microbes and the phytopathogens displayed a competitive rate during in vitro and in vivo activity, and likewise contributed to plant growth promotion. Furthermore, phages’ negative impact has skyrocketed due to certain bacteria species that produce antibiotics (*Bacillus thuringiensis*) as a result of the coevolution of phage resistivity to antitoxin produced by the bacteria [70]. There are diverse effects produced by phages in the rhizosphere soil when mutation occurs in microbial diversity, the functional diversity of these microbes, and the cycling of nutrients through the process of the biogeochemical cycle [71].

Besides phages, certain microbes called protists (amoeba, paramecium, euglena, etc.) do contribute to rhizosphere ecosystem coevolution via selective benefits to bacterial genotypes that prevent predation [72]. The coevolution of the act of preventing predation could likewise prevent competition between the bacteria and the protists, so that when toxic substances (secondary metabolites) are induced by bacteria, they would destroy the existence of the protist. The coevolution of anti-protist defenses, which are required direct defenses, and the trophic level of microbial interaction can carry out essential functions in their ecosystem (rhizosphere soil), and result in the coevolution of plant–microbial interaction.

**Figure 1:**
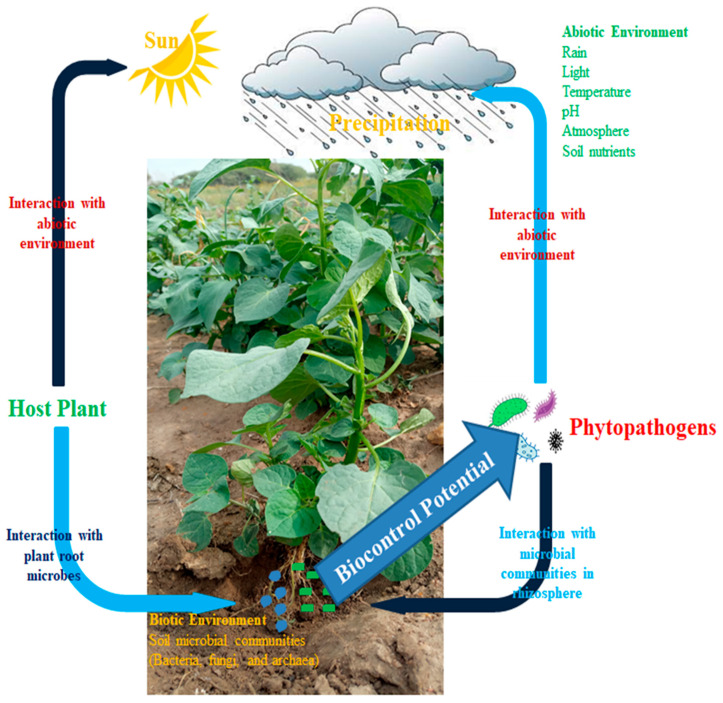
Plants influence soil microbial communities in plant–microbe interactions.

Figure 1 reveals the interaction of beneficial and pathogenic microbes. This occurs due to plant health status, which was a result of the availability of sunlight for photosynthesis and water for transpiration. Due to plant health, its roots produced root exudates which attract beneficial microbes inhabiting the surrounding soil to migrate into the rhizosphere soil of the plant to feed on the carbon substrates of the exudates. The plant also provides shelter for these microbes, and in return, the microbes carry out specific functions which include pathogen or disease resistance, improving plant health status, and mitigating abiotic stresses affecting the plants.

The figure explains how the green plant manufactures its food substance from sunlight by photosynthesis. The arrow shows the interaction of the host plant with the abiotic factors, sunlight and rain; i.e., plants interact with abiotic conditions to make inorganic food substances.

The arrow pointing to the soil explains how the host plant produces root exudates to attract beneficial microbes into the rhizosphere soil, thereby involving symbiotic association between plants and microbes. The arrow pointing from the soil to pathogens explains the biological control activity of beneficial microbes against pathogenic microbes, while the arrow from pathogenic microbes to soil shows the invasion of pathogenic microbes to destroy the plant. Unknowingly, beneficial microbes act as a defense mechanism against pathogenic microbes’ potential. The interaction between beneficial and pathogenic microbes explains the concept of coevolution, whereby pathogenic microbes mutate from their original form to escape beneficial microbe control, yet beneficial microbes strategize their mechanisms of attack to inhibit the incidence of pathogenic microbe attack (microbial arms race).

## 4. Plant Holobiont Potential to Promote Sustainable Agriculture

Microbial study has gained the interest of researchers because of the significance of microbial communities in agricultural practices, biotechnological development, medicine, food production, drugs, cosmetics, etc. Despite the microbiota inhabiting the soil fauna’s significance to agriculture by promoting aesthetic values, including plant growth promotion, and disease-resistant abilities, among other factors (Figure 1), the study of soil fauna gut microbiota still requires more innovation. Comparison between rhizosphere microbes and soil fauna gut microbes has been conducted in various research [73,74]. The similarity between these organisms is that they are both beneficial to their host in terms of health and survival. As a matter of fact, rhizosphere microbiomes are identified for their advantageous effects on crops’ health status and their development. Plants develop various methods to interchange chemical signals with microbial communities, with benefits including advantageous microbial functions like nutrient acquisition, carbohydrate metabolism, etc.; preventing plants from phytopathogen invasion; and biocontrol potential against phytopathogens [75].

The co-evolution of microbes leads to dynamic changes in their interactions that ultimately enhance plant health. Through long-term evolutionary pressures, beneficial microbes adapt by developing improved mechanisms for nutrient exchange, stress tolerance, and signaling that strengthen symbiotic relationships with plants [76]. At the same time, plants evolve to better recognize and accommodate these beneficial microbes while resisting pathogens [77]. This reciprocal adaptation refines microbial communication, cooperation, and competition within the rhizosphere, promoting a balanced microbiome that supports plant growth, immunity, and resilience against environmental stressors [78]. According to the study of Mukherjee, et al. [79], plants are regarded as the most excellent holobionts that associate and house phytomicrobiomes and other related cellular organelles like chloroplasts and mitochondria, which are necessary for the survival of plants. The coevolution of microorganisms has produced interactions between plants and microbes in agricultural ecosystems [80]. New inventions should be employed to uphold agricultural systems and improve the ability to produce abundant harvests. Orientations should be conducted regarding phytopathogen-resistance-breeding events. Biotechnologically, crops have been produced with some pathogen-attack-resistant characteristics (ancient or wild relatives) [81]. A typical example of such a plant is Bt corn. The plant already produced biotechnologically can elicit toxic substances that can rupture the gut of insects trying to feed on the plants or prevent the growth of phytopathogens (Figure 1). The existence of these new species of corn is a result of evolution in plants and the adaptation of such plants to the environment.

Some of the plant-disease-resistance characteristics selected are related to metabolized crop modifications, while others are products of interactions that promote biological control activities carried out by the diversity of microbes inhabiting the plant or rhizosphere soil. Even though the reproduction process has been flourishing among these beneficial microbes, resistance abilities arise within a specific time after a new generation of microbes has been produced as a result of replication. The circumstances are linked to a variety of processes, like the coevolution of phytopathogens that possess virulence traits in plants. Some methods depend on the generation of crop varieties having phytopathogen resistance traits, which reveal controversial effects upon phytopathogen invasion. The question to investigate here is how the evolution of these resistance plants is different from plants of the same species that have no resistance attributes against phytopathogens. The answer is a result of the coevolution of antitoxin-producing microbes that are beneficial to the plants and live in the plant environment as either rhizosphere organisms or endophytes (i.e., organisms living within the plant tissue) [28]. The biocontrol activities of these organisms makes them stand out against spoilage organisms and contribute to plant growth, and evolution results in changes in the plant species from being without antitoxin effects to being plants with antitoxin effects [82]. Therefore, a new generation of plants produces a new generation of fruits that develop faster, avoid spoilage, and maintain shelf life during germination, harvest, and the post-harvest phases [83]. The process of applying unrelated microbes to agricultural land to inhibit or eradicate the existence of phytopathogens can assist in monocultural farming. Utilizing plant breeding methods with technology involving beneficial microbe formulation (biofertilizers, biopesticides, biostimulants, etc.) would help induce immune responses in plants against pathogens [84].

Microbial communities dwelling in the rhizosphere soil of plants are advantageous and react to the ecosystem. Diverse species frequently occur in the soil, including PGPR, PGPF, archaea, nematodes, arbuscular mycorrhizae, and other beneficial microbes [1]. Each species exists in abundance in the soil and plants, also known as endophytes in the case of phytomicrobiomes. The diversity of microbiomes interacting with plants is different among the cultivars of microbial species, while some cultivars are of the same species. The microbial diversity can be affected by various factors, including soil pH, moisture content, soil type, water retention ability, soil texture, and other factors relating to soil, like nutrient availability [17]. Among the variety of microbiomes inhabiting the rhizosphere, researchers have studied a few species to observe their beneficial associations with a few plants. The inference reported from the study shows that there are lots of interactions that have not been quantified. Essentially, one of the factors leading to the evolution of plant species resulted from interaction with phytopathogens and parasites as a result of parasitic association, which affects plant growth and reduces crop yield. Symbiotic association between the plants and beneficial microbes tends to destroy the parasitic association; therefore, new varieties of plants evolve [85]. They can either possess the potential to respond against attack by inducing an immune response against the parasites, resist the parasitic invasion, or they may learn to survive and produce their crops despite the attack from parasites. A common example of symbiotic association observed with the root nodules of leguminous plants and arbuscular mycorrhizal fungi [86] explained how the population density of phytomicrobiomes is important for plant growth. The complex interactions take place in the rhizosphere soil of plants, which results in coevolution of microbial diversity associating with plants [87]. The physiological state of plant–microbe interaction has doubled up via the coevolution of the microbes as a result of their dependent states, while microbes undergoing independent association go into extinction due to a reduction in microbial growth.

### 4.1. Comparative Findings and Active Controversies

Although there are still significant differences about causality and scope, recent research generally agrees that microbiome diversity frequently corresponds with better plant health. In many crops, microbiome richness and pathogen prevalence are negatively correlated, according to large-scale surveys. However, targeted inoculation trials occasionally fall short of reproducing these protective benefits in the field, indicating context dependency. While some researchers view these discrepancies as proof that variety in and of itself provides protection for plants (a buffering effect), others contend that protection is dependent on certain functional taxa or keystone strains whose actions are influenced by host genotype and environmental circumstances. Linked controversies include whether the holobiont should be treated as a selectable unit (selection-on-host-plus-microbiome) or whether selection primarily operates on individual partners, and how broadly results from model systems (Arabidopsis, tomato) generalize to diverse crop species and soils. A balanced treatment should present both sides and stress that resolving these debates requires experiments that combine taxonomic, functional, and evolutionary perspectives across scales.

### 4.2. Methodological Limitations of Culturing, Metagenomics, and Inference

Interpretations are influenced by the advantages and disadvantages of each main approach to the study of plant–microbe coevolution. Broad taxonomic and functional snapshots are provided by shotgun metagenomics [5]; nonetheless, this method has drawbacks, including biases in DNA extraction, insufficient reference databases that restrict taxonomic precision for many soil microorganisms, and difficulties differentiating between dormant and active cells. When gene expression and ecological environment are disregarded, functional inference, which is the process of predicting metabolic pathways from gene presence, can be misleading. Relative-abundance patterns are provided by amplicon sequencing (16S/ITS) [39], but strain-level resolution is essential for identifying intraspecific variation, and recent coevolutionary changes are weak.

Culture-dependent techniques recover live isolates, which makes mechanistic studies (genomic analysis, phenotyping, and experimental evolution) possible. However, a limited, nonrepresentative percentage of the microbiome is usually captured by culture bias. Although multi-omics, including metagenomics, metabolomics, metatranscriptomics, and metaproteomics, aid in bridging identity and function, they are expensive, analytically demanding, and susceptible to sample procedures [88]. Lastly, a lot of research uses single-timepoint sampling, which masks the temporal dynamics that are essential to coevolution. It will be easier for readers to understand conflicting results from various studies if these tradeoffs are clearly stated.

### 4.3. Experimental Design Limitations and Inference Challenges

Without concrete proof of reciprocal fitness effects, comparative and correlational studies frequently extrapolate selection or coevolution from patterns such as phylosymbiosis or host genotype and microbiome covariation. On the other hand, whereas microcosm and laboratory evolution studies can show quick reciprocal adaptation, they usually employ artificial media, single-stressor regimes, or simplified communities that amplify certain processes while stifling the ecological interactions seen in soils. Furthermore, a large number of field microbiome investigations are not sufficiently replicated across seasons and habitats to distinguish heritable evolutionary change from temporary ecological adjustments. Identifying these inferential gaps can help make a stronger case for the areas that need more attention in future research.

### 4.4. Translation of Laboratory Evolution Field

Microcosms, serial transfer, and synthetic communities are examples of laboratory evolution that are effective for demonstrating mechanisms. They may recognize certain genes that are being selected for, exhibit arms-race dynamics, e.g., phage–bacteria, and adapt quickly [89]. However, direct translation is constrained by several factors, which include the facts that community complexity and emergent interactions explain how hundreds to thousands of species interact in the wild, and adaptations seen in paired-lab experiments can be neutralized or amplified by emergent features, including cross-feeding, facilitation, and indirect impacts. Environmental heterogeneity is another factor, in which actual fields, nutrient patches, moisture variations, soil structure, and microclimates produce spatially diverse selection pressures that are not present in well-mixed laboratory systems. Other factors are as follows.

Seasonality and temporal scale: In nature, coevolution takes place throughout host generations and seasons. While many laboratory trials span tens to hundreds of microbial generations, they are unable to replicate long-term demographic dynamics or seasonal host phenology. Host genetic and phenotypic variety: Single plant genotypes are frequently used in lab experiments; in agricultural areas, microbial recruitment and selection are influenced by crop genetic diversity, root architecture, and farming techniques.

Horizontal gene transfer and mobile elements: Unlike sterile lab microcosms, mobile genetic elements (plasmids, phages, and transposons) may transmit adaptive characteristics quickly in the field. Their influence and pace vary between controlled and open systems.

However, under field circumstances, promising lab-evolved inoculants sometimes lose their effectiveness due to these mismatches. The field requires multi-season repeated field trials, intermediate-scale mesocosm studies, and “evolution-aware” inoculant testing that monitors community reassembly, horizontal gene flow, and persistence under realistic management regimes to enhance translation.

## 5. Recommendations to Reduce the Knowledge Gap and Improve Inference

- Connect taxa, genes, and activities, and combine multi-omics, strain-resolved genomics, and culture;
- Determine whether kinds of interactions scale to increasingly complex systems, and use artificial communities with varying levels of complexity;
- Maintain experimental control while conducting long-term, repeatable field studies using mesocosms that provide ecological and seasonal realism;
- Quantify reciprocal fitness effects across host genotypes and settings, conduct experimental evolution and reciprocal transplant trials in soil;
- Assist the community in identifying the environmental or managerial factors that impact success, report metadata, and negative outcomes when inoculants fail.

### 5.1. Practical Implications and Future Direction

Biofertilizer and Biocontrol Design: Our understanding of the coevolution of microbes and plants changes our conception of biological control agents and biofertilizers. Conventional products sometimes depend on a single “elite” strain, such as *Bacillus subtilis* or *Rhizobium*, which works well in greenhouse experiments but becomes ineffective in the field due to its inability to compete with indigenous bacteria and adjust to shifting soil conditions. Instead, synthetic consortia formed to resemble naturally coadapted microbial networks are preferred from a coevolutionary viewpoint. Such consortia might provide multi-layered functional redundancy by combining nitrogen-fixing bacteria with phages that restrict pathogen abundance and fungi that mobilize phosphorus. Each member is selected based on characteristics that have been shown to evolve in tandem with the host: chemotaxis toward particular root exudates, compatibility with other members of the community in terms of quorum sensing, and the generation of chemicals that either outcompete or discourage pathogens. While experimental evolution can pre-adapt bacteria to local soil pH, moisture, or temperature profiles, genomic and transcriptomic screening finds alleles linked to long-term rhizosphere survival. Anticipating the counter-adaptation of infections, such as the development of phage resistance or the enzymatic detoxification of antimicrobial chemicals, is equally crucial. Such evolutionary foresight informs regulatory criteria for safe field deployment and lowers the danger of effectiveness breakdown across several planting seasons.

### 5.2. Disease-Resistant and Microbiome-Responsive Breeding

Single-gene crop breeding has frequently resulted in “boom-and-bust” cycles, in which diseases adapt to get past plant defenses. A more comprehensive approach is suggested by the recognition of the plant as a holobiont, or a host plus its microbiome, which opts for genotypes that reliably attract beneficial microbiota. For example, certain tomato lines release organic acids that encourage bacteria that solubilize phosphate, while other sorghum kinds release phenolic compounds that enrich Actinobacteria that can inhibit Fusarium wilt. These characteristics are heritable and genetically changeable. With genome-wide association studies, breeders can now link specific loci to root exudate profiles, rhizosphere composition, or the stability of key microbial partners across environments. Marker-assisted selection and genomic prediction can therefore incorporate microbial recruitment as a breeding target alongside yield or drought tolerance. By fostering plant genotypes that maintain a coevolved microbial shield, breeders create crops whose disease resistance is dynamic and adaptable, reducing reliance on chemical pesticides and extending the effective life of resistance traits.

### 5.3. Climate Resilience and Stress Adaptation

Extreme heat, excessive rainfall, and soil salinization are some of the many pressures brought on by global climate change that pose difficulties for both plants and their symbionts. Long-standing coevolution in harsh environments is frequently the source of microbes that improve abiotic-stress tolerance. For example, endophytic fungi from alpine grasses provide cold and UV tolerance, halophilic bacteria isolated from salt marsh plants improve ion homeostasis in their hosts, and desert cyanobacteria produce extracellular polysaccharides that improve soil moisture retention. By identifying these co-adapted microbiomes, beneficial gene clusters may be transferred or specifically inoculated into crops cultivated in similarly demanding environments. Maintaining the whole interaction network is essential because stress-resistance features are often polygenic and entail complicated signaling, including osmoprotectant production, reactive oxygen species scavenging, and abscisic acid pathways. As the climate continues to change, multi-season monitoring is required to make sure that imported communities stay stable. Furthermore, anticipatory management strategies, such as pre-conditioning soils with microbial consortia that are known to withstand a wide variety of temperature and moisture regimes, can be informed by knowledge of how warming changes selection pressures on the host and microbiome.

### 5.4. Emerging Technologies Enabling These Applications

Multi-omics developments offer a previously unheard-of level of clarity on the evolutionary interactions between microbes and plants. While metatranscriptomics and metaproteomics show which genes are actively expressed in the field, documenting host–microbe communication in real time, metagenomics reveals community members and their genetic potential. Rare taxa that may serve as keystone species despite their low abundance are discovered using single-cell genomics. When combined with metabolomics and isotope tracking, these methods provide a direct connection between microbial activity and plant physiology and nutrient flows. Chemostats and microfluidic “rhizosphere-on-a-chip” devices are examples of high-throughput experimental evolution systems that enable researchers to apply controlled selection pressures, such as varying root exudates or drought cycles, to observe adaptation across hundreds of generations. In order to investigate how interactions scale as variety increases, Synthetic Community (SynCom) engineering reconstructs natural consortia of increasing complexity, bridging the gap between lab and field. Precision agriculture may be guided by machine-learning models trained on this data, which can forecast how particular plant genotypes and management techniques will alter the makeup and function of the microbiome.

### 5.5. Agricultural Application and Future Direction

A coevolutionary viewpoint offers a path forward for sustainable agriculture of the future. By favoring plant genotypes that attract and sustain advantageous microbial consortia, breeding programs can efficiently select for durable holobionts rather than isolated plants [90]. Precision biofertilizers, biostimulants, and phage-mediated biocontrol agents are examples of microbiome-based technologies that use coevolved features to improve nutrient absorption and inhibit infections without the environmental costs associated with synthetic agrochemicals. The development of prediction models that relate host genotype, microbiome composition, and environmental factors to crop performance is now made possible by multi-omics platforms, including metagenomics, transcriptomics, and metabolomics [91]. By combining these methods with extended field experiments, it will become clearer how stresses brought on by climate change, such as drought or salt, alter the evolutionary paths of plants and microbes. In the end, treating crops as dynamic holobionts rather than static genotypes provides a potent method for creating robust, low-input agroecosystems that can continue to produce food for the world in the face of constantly shifting environmental conditions.

## 6. Conclusions

One of the primary forces behind the resilience of agriculture and the functioning of terrestrial ecosystems is the coevolution of plants and the microbial communities that accompany them. Plant–microbe relationships are dynamic, reciprocal processes influenced by continuous natural selection, as this review shows by combining molecular, ecological, and evolutionary data. In order to improve plant fitness and production, mutualistic and antagonistic coevolution have an impact on nutrient cycle, stress tolerance, and disease resistance. Understanding plants as holobionts integrating parts of their hosts and their microbiomes provides a conceptual foundation for creating agricultural systems that are both sustainable and evolution-aware. Coevolutionary mechanisms, precision biofertilizers, and microbiome-based biocontrol techniques that improve crop performance under environmental stress may all be guided by the application of coevolutionary concepts. Characterizing intraspecific microbial variation, using multi-omics techniques to connect genetic and ecological functions, and translating laboratory results into long-term, field-based experiments should be the main goals of future study. A better comprehension of these evolutionary mechanisms will make it possible to create low-input, robust agroecosystems that can maintain global food security in the face of climate change.

## Figures and Tables

**Table 1:** Natural selection contributes to the speciation and diversification of microbial mutation and recombination.

Microbes	Microbial Functions	Natural Selection	Reference
Bacteria species	The speciation of microbes is controlled by divergent natural selection, which contributes to gene flow and reproductive modes.	This occurs either genetically or ecologically.	[40]
Cyanobacterium (*Microcoleus*)	The bacteria produce certain genes that control secondary metabolite synthesis and stress response.	The bacteria are responsible for at least 12 lineages of the global speciation continuum.	[41]
Endophytes	These microbes are more involved in phylosymbiosis signaling compared to other microbes, like rhizosphere microbes and epiphytes.	The phylosymbiosis is attributed to the microbial phylogeny and taxonomic classification of the plants they interact with.	[42]
Pathogenic bacteria	The study presents how the bacteria colonize and proliferate within or between the host plant(s) during the process of bacterial phylogeography.	The population of the bacteria is controlled by genetic drift, mutation, recombination, natural selection, and demographic history.	[43]
Phytopathogen (*Pseudomonas syringae*)	Plants produce a resistance gene (R-gene) against the invasion of the phytopathogen.	This reveals the similarity clustering, with eco-evolutionary dynamics arising from the ecological niche due to the coexistence of the bacterial strain.	[44]
Endosymbiotic bacteria (*Sodalis*)	The bacteria are responsible for gene production that carries out functions such as amino acid biosynthesis, including the respiratory chain and DNA repair pathways.	Natural selection involves the coming together of various functions between the microbes found in the symbiont lineage and stochastic mutation.	[45]
Symbionts and insects	The symbiont is involved in a beneficial interaction with the insects.	They contribute to insect response to various stresses in their ecosystem, and by so doing, help insects adapt to various environments.	[46]
DNA and Mitochondria	There is transmission of plastid DNA to mitochondria present in the green leaves of plants.	The rate of evolution of the genetic materials depends on the high contingency of mitochondrial genomic evolution.	[47]
Interspecies microbes (*E. melliodora* and *E. sideroxylon*)	The organisms can produce genetic materials that look like eucalyptus plants to recognize structural variation.	Structural variation reveals the differences between the rate of recombination and genetic differentiation between the two interspecific interactions.	[48]

## Data Availability

Not applicable.

## Abbreviations

UV	Ultraviolet
DNA	Deoxyribonucleic Acid
PGPR	Plant Growth-Promoting Rhizobacteria
PGPF	Plant Growth-Promoting Fungi
